# Association of race, ethnicity, and housing stability with COVID-19 testing method by investigators in underserved populations 2020–2023

**DOI:** 10.3389/fpubh.2025.1605167

**Published:** 2025-09-24

**Authors:** Edwin Wilbur Woodhouse, Timothy Veldman, Elizabeth Lydon, Lisa Wruck, Elizabeth Petzold, Paul Drain, Eyal Oren, Susan M. Kiene, Corinne McDaniels-Davidson, Lisa Gwynn, Krista M. Perreira, Barrie Harper, L. Gayani Tillekeratne, Susanna Naggie, Micah T. McClain, Christopher W. Woods

**Affiliations:** ^1^Division of Infectious Diseases, Duke University School of Medicine, Durham, NC, United States; ^2^Duke Clinical Research Institute, Durham, NC, United States; ^3^Durham Rescue Mission, Durham, NC, United States; ^4^Departments of Global Health, Medicine, Epidemiology, University of Washington, Seattle, WA, United States; ^5^Division of Epidemiology and Biostatistics, San Diego State University, San Diego, CA, United States; ^6^School of Public Health, San Diego State University, San Diego, CA, United States; ^7^Miller School of Medicine, University of Miami, Miami, FL, United States; ^8^Department of Social Medicine, School of Medicine, University of North Carolina at Chapel Hill, Chapel Hill, NC, United States

**Keywords:** underserved populations, COVID-19 testing, community health, disparities, PCR, antigen

## Abstract

**Background:**

Expanding SARS-CoV-2 testing was a critical part of community-based health efforts during the COVID-19 pandemic. In the RADx-UP consortium, a large NIH-funded network of community-engaged researchers in the United States, investigators were able to choose between PCR- and antigen-based testing strategies in community-based research settings. Data analyzing how COVID-19 diagnostics are chosen and utilized in research of vulnerable and underserved populations is limited.

**Objectives:**

To examine the association of race, ethnicity, and housing stability with a PCR- or antigen-based testing strategy within COVID-19 testing projects in the RADx-UP consortium.

**Methods:**

Testing protocols and investigator survey data describing target populations for community-engaged research projects were analyzed for association between race, ethnicity, and housing stability with SARS-CoV-2 test type. Community-engaged research projects were included if they were funded and approved to use PCR- and/or antigen-based COVID-19 testing by the RADx-UP testing core between 2020 and 2023. Multivariable adjustment to assess for confounding was then performed using rurality, project size, pandemic phase, and census region.

**Results:**

Sixty-seven projects (representing 479,410 participants) were included in the analysis. Overall, 24 (36%) projects chose an antigen-only testing strategy compared to 43 (64%) that chose a PCR-based strategy. No significant differences in distribution were seen in inclusion of PCR-testing by race (16 of 21 for Black race versus 27 of 46 for non-Black race, *p* = 0.198), ethnicity (22 of 33 for Hispanic ethnicity versus 21 of 34 for non-Hispanic ethnicity, *p* = 0.765), or housing stability (10 of 17 for unstable housing versus 33 of 50 for stable housing, *p* = 0.728) within intended population.

**Conclusion:**

Race, ethnicity, and housing stability of an underlying vulnerable population was not significantly associated with the decision by community investigators regarding which COVID-19 testing strategy was most appropriate. Future research efforts should remain vigilant to offer emerging diagnostic technologies in the most equitable and appropriate ways.

## Introduction

Early rollout of COVID-19 testing efforts in the United States exposed disparities in access to testing, with Black and Hispanic communities in the United States among the most inequitably affected (1). The earliest months of the COVID-19 pandemic were characterized by widespread testing shortages and long wait times for results, which improved as rapid PCR and antigen tests became available (2). Rapid Acceleration Diagnostics for Underserved Populations (RADx-UP) represented the single largest investment in health disparities and community-engaged research in National Institutes of Health (NIH) history. A total of 137 projects and 25 Rapid Pilot Projects comprise the RADx-UP program funded by the NIH, and included community engaged partnerships with academic institutions, community-based organizations, federally qualified health centers, and historically under-represented colleges and universities (3). This cross-consortium of community-engaged research projects had the goal of increasing access to COVID-19 testing in underserved populations, assessing which diagnostic options were most acceptable and in what setting, and evaluating which testing interventions were most effective to decrease related disparities (4). The recruited population characteristics and methods used to reach these people varied. (see Supplementary Appendix). As part of this effort, improving education about, access to, and utilization of COVID-19 testing was fundamental to addressing health disparities and inequalities within underserved populations (5).

A decision often faced by community leaders and principal investigators (PI) was in choosing between nucleic acid amplification testing (most commonly PCR-based) and rapid antigen-based testing. Although PCR-based testing holds improved test accuracy over antigen-based testing, it is more technically challenging and can be difficult to implement on a community level due to a longer time-to-result, requires technical expertise, is more expensive, and may remain positive after clinical recovery (6). PCR-based diagnostics are considered the gold-standard for initial detection of SARS-CoV-2 and other respiratory viruses, but are comparatively unavailable or too expensive to be performed on a community level at the point-of-care. Antigen-based COVID-19 tests can be performed in a matter of minutes, are inexpensive, and can be performed and interpreted with little-to-no expertise. However, the convenience of antigen testing comes at the expense of lower test accuracy, with less than 20% sensitivity before symptom onset, and a greater than 30% decrease in overall test sensitivity when compared to PCR-testing (7, 8). Throughout the pandemic testing options for researchers and participants changed over time, particularly as rapid at-home antigen tests became more widespread (9, 10).

Academic and community investigators in the RADx-UP consortium were frequently faced with the decision on how to best implement COVID-19 testing and how to grapple with the tension of choosing between antigen or PCR-based testing strategies in a way that alleviated health disparities in underserved populations. This forced decision process created a natural registry of community-based investigator preferences, which can be analyzed to better understand how community-based researchers approach similar situations, particularly within infectious disease testing programs.

Investigators of RADx-UP projects were required to submit testing strategies to a central testing core, which was comprised of a dedicated team of clinicians, microbiologists, and public health experts familiar with COVID-19 diagnostics (11). Investigators were encouraged to use what they felt was the most appropriate testing strategy for their local targeted community. Testing protocols were approved if they were scientifically sound based on a standardized Testing Assessment Quality Management Tool while following US Food and Drug Administration (FDA) emergency use authorization (EUA) guidance (12). Size and breadth of funded interventions varied, from randomized controlled trials of behavioral interventions to questionnaires about vaccine willingness among children or vulnerable populations (13–15). Community partnerships were required for all interventions but varied in scope and scale by individual projects.

We aimed to describe the choice of antigen- or PCR-based COVID-19 testing strategies to reach underserved populations by community investigators within the RADx-UP program, and specifically to analyze associations of PCR- and antigen-based testing strategy by race, ethnicity, and housing stability of intended populations between 2020 and 2023. As community-based research intends to understand and correct social underpinnings of health, understanding which testing strategy was selected in practice could be informative for future testing and disease prevention efforts.

## Methods

Data from all participating projects within the RADx-UP consortium were collected using standardized common data elements (CDE), as well as standardized surveys of investigators. To appropriately combine data from disparate study designs and collection strategies, the RADx-UP Coordination and Data Collection Center (CDCC) collected Project Structure Metadata and Study Design information for each aim in a study’s protocol via survey from project managers and project investigators with support from program officers for aim-related data. This survey collected information on a study’s primary target population, which was defined as the underserved population that the project intended to research and engage in context of public health and disease prevention, and which generalizations from the study sample were to be made. Data on race and ethnicity were defined and collected in accordance with current guidance for race and ethnicity from NIH (16). This survey also collected a study’s secondary target population, which included the same variables as primary target population but was defined as underserved populations that were represented in their study population but were not intentionally targeted and not of primary interest. Project investigators were instructed to choose all that applied from a list of underserved populations eligible for RADx-UP funding provided by the NIH. These datasets and survey information were combined with internal data from RADx-UP testing core describing a chosen testing modality, any modifications to testing strategy, and the date of testing strategy approval. Projects were included if they submitted a selected COVID-19 testing assay and were approved by the RADx-UP Testing core, which reviewed that each project met FDA and NIH requirements for COVID-19 testing. Projects were excluded if they used 50% or more antibody testing or did not clearly describe intended COVID-19 testing modality in their protocol.

### Data analysis

The study population of this analysis included projects that had submitted data to the RADx-UP core analytic dataset, which was required of all projects. Projects that were deemed non-testing projects, did not receive funding for COVID-19 testing from RADx-UP and therefore were outside purview of RADx-UP technical guidance, or utilized testing strategies that were antibody-based were excluded. Testing protocols were then designated “antigen” or “PCR-based” strategies based on protocol review. Projects that used combined antigen and PCR-based testing were classified as “PCR-based” due anticipated low number of projects using both and high rates of confirmatory PCR-testing for participants within those projects using both.

Main exposure variables were defined based on investigator survey data on target population for race, ethnicity, and housing stability. Based on the study design survey to investigators from RADx-UP, race was defined as African American/Black; Asian, Native Hawaiian and Pacific Islanders; Alaska Natives/American Indian; and/or none of the above. Ethnicity was defined as Hispanic or non-Hispanic, and unstable housing was defined as low-income housing or homelessness. Principal investigators were asked via survey if their intervention targeted one or more of these populations and were allowed to choose more than one response for exposure variables as applicable. Full details on definitions of exposure variables can be found in the Supplementary Appendix. All core data was held and analyzed by the Coordination and Data Collection Center, who advised and performed statistical support throughout the RADx-UP Consortium.

Analyses were conducted at the project level (as opposed to participant level). For primary analysis, indicator variables were derived from the primary target population listed in the RADx-UP Study Design Metadata, as described above. The exposures were defined by the primary target population, then analyses were repeated for secondary target population. Counts and percents of testing strategy were calculated within level of project primary target population (race, ethnicity and housing stability) and other project characteristics. Barnard’s exact test was used to compare distributions of testing strategy (antigen only vs. inclusion of PCR) separately within level of race, ethnicity, and housing stability. Levels of primary target population were described and tested in separate models because levels were not mutually exclusive, i.e., investigators were able to choose more than one target population for each project. Barnard’s exact test was selected to improve power given relatively small number of projects, low cell counts of certain exposure groups and non-parametric distributions. Multivariable logistic regression was performed to assess for differences in testing strategy by race, ethnicity, and housing stability when adjusting for pre-specified potential confounders that included project size, geographic region, rurality, and phase of pandemic as an exploratory analysis due to low event counts. Statistical threshold of 0.05 was used to determine significance throughout this analysis. To ensure stability of models only groups with at least 10 events were included. Analyses were performed using SAS 9.4 at Duke Clinical Research Institute (by authors EL and LW) and reported independently to writing team. All projects were approved by local Institutional Review Boards (IRB) and this cross-consortia analysis was approved by an umbrella IRB protocol at Duke University.

## Results

At time of analysis on August 15, 2024, 114 of 137 total projects had data available in the core analytic data set, which included 1,032,720 participants. Forty-one projects were excluded because they were non-testing projects or testing was not funded and supervised through RADx-UP programs. Six additional projects were excluded due to antibody testing being the primary testing modality. Sixty-seven community-based projects, representing 479,410 participants, were included in the final analysis (Supplementary Figure 1). Twenty-four projects used an antigen-only testing strategy and 43 projects used a PCR-based testing strategy. Within the 43 PCR-based testing projects, nine used a combined antigen and PCR testing strategy, with high rates of confirmatory testing in these cases. All included projects had demographic data available on intended target population of their study, and the three exposure variables.

The full distribution of intended testing modalities is seen in Table 1. Race, ethnicity, housing instability, and rurality were all tested separately with Barnard’s exact test and no differences were statistically significant. PCR-based testing strategies were more common in all categories. Antigen-only testing strategies were more common in later phases of the pandemic, with 11/19 (57.9%) projects that were begun during Omicron phase (November 2021 and beyond) choosing antigen-only, compared to 11/42 (26.2%) of projects that begun during the Alpha wave (November 2020 to May 2021) who selected antigen-only testing methods.

**Table 1:** Distribution of project testing strategies.

Characteristic^0^	Overall *N* = 67	Antigen only *N* = 24 (35.8%)	Inclusion of PCR *N* = 43 (64.2%)	*p*-value^3^
Black				0.198
Yes	21/67 (31.3%)	5/21 (23.8%)	16/21 (76.2%)	
No	46/67 (68.7%)	19/46 (41.3%)	27/46 (58.7%)	
Asian, native Hawaiian or Pacific Islander				0.884
Yes	7/67 (10.4%)	3/7 (42.9%)	4/7 (57.1%)	
No	60/67 (89.6%)	21/60 (35.0%)	39/60 (65.0%)	
Alaska natives/American Indian				0.572
Yes	5/67 (7.5%)	1/5 (20.0%)	4/5 (80.0%)	
No	62/67 (92.5%)	23/62 (37.1%)	39/62 (62.9%)	
Hispanic ethnicity				0.765
Yes	33/67 (49.3%)	11/33 (33.3%)	22/33 (66.7%)	
No	34/67 (50.7%)	13/34 (38.2%)	21/34 (61.8%)	
Housing instability^1^				0.728
Yes	17/67 (25.4%)	7/17 (41.2%)	10/17 (58.8%)	
No	50/67 (74.6%)	17/50 (34.0%)	33/50 (66.0%)	
Rural				
Yes	7/67 (10.4%)	2/7 (28.6%)	5/7 (71.4%)	
No	60/67 (89.6%)	22/60 (36.7%)	38/60 (63.3%)	
Region				
Northeast	10/67 (14.9%)	4/10 (40.0%)	6/10 (60.0%)	
Midwest	11/67 (16.4%)	4/11 (36.4%)	7/11 (63.6%)	
South	19/67 (28.4%)	6/19 (31.6%)	13/19 (68.4%)	
West	18/67 (26.9%)	9/18 (50.0%)	9/18 (50.0%)	
Multiple region or other US territory	9/67 (13.4%)	1/9 (11.1%)	8/9 (88.9%)	
Project size (categorical)				
<280	17/67 (25.4%)	9/17 (52.9%)	8/17 (47.1%)	
280 to <837	17/67 (25.4%)	7/17 (41.2%)	10/17 (58.8%)	
837–2,607	17/67 (25.4%)	2/17 (11.8%)	15/17 (88.2%)	
2,607+	16/67 (23.9%)	6/16 (37.5%)	10/16 (62.5%)	
Phase of pandemic^2^				
Alpha wave	42/67 (62.7%)	11/42 (26.2%)	31/42 (73.8%)	
Delta wave	6/67 (9.0%)	2/6 (33.3%)	4/6 (66.7%)	
Omicron and beyond	19/67 (28.4%)	11/19 (57.9%)	8/19 (42.1%)	

^0^The primary analysis derives indicator variables from the Primary Target Population listed in the RADx-UP Study Design Metadata based on project protocols; intended Test Type is based on selected assay submitted to RADx-UP Testing Core. ^1^Unstable housing characterized in the models as a project focused on either homelessness or low-income housing as the target population. ^2^Phase of pandemic is based on the date the project was greenlit by the RADxUP Testing Core. ^3^*p*-value for Barnard’s exact test of independence.

No significant differences were seen, and no effect size changed direction, after adjustment for potential confounders for likelihood of PCR-based testing by race, ethnicity, or housing stability. Unadjusted and adjusted odds ratios of intended testing modalities are shown in Table 2. Intended testing strategy by secondary target population was analyzed and not found to be different from primary population results. Full descriptions of all included RADx-UP projects, the observed population characteristics by test type, and a comparison of primary target versus observed study population demographic characteristics can be found in the Supplementary material.

**Table 2:** Relative odds of project PCR-based testing strategy by race, ethnicity, and housing stability.

Unadjusted and multivariable adjusted odds ratios of intended testing modality by exposures				
Exposure	Unadjusted^1^		Adjusted^1^	
	OR (95% CI)	*p*-val	OR (95% CI)	*p*-val
Race^2^				
Black	2.25 (0.70, 7.21)	0.171	3.21 (0.64,16.13)	0.156
Hispanic ethnicity	1.24 (0.45, 3.37)	0.676	2.23 (0.59, 8.41)	0.239
Unstable housing^3^	0.74 (0.24, 2.28)	0.595	0.60 (0.12, 3.07)	0.543

^1^Multivariable adjusted model estimates examine the association between each of the main exposures and COVID-19 Testing Modality, adjusted for potential confounders: rurality, project size, pandemic phase and region. ^2^Race- only Black race had event rates sufficient for logistic regression. ^3^Unstable housing is defined as a project focused on either homelessness or low-income housing as the target population.

## Discussion

The COVID-19 pandemic exacerbated and exposed health inequalities in the United States, and the RADx-UP consortium was the largest NIH-funded effort of its kind to understand and address health disparities (17). Community-based health research partnerships are intended to understand and address these issues (18). In the context of COVID-19, a fundamental decision faced by researchers was choosing between easy and convenient point-of-care rapid antigen tests and highly accurate but more technical PCR-based strategies. This study examined investigators’ choice of antigen versus PCR-based testing strategies within the largest consortium of community-based research in underserved populations, and did not find any significant association between testing strategy and race, ethnicity, or housing stability of the target population. Additionally, exploratory modeling was performed to assess for project size, rurality, and geography as possible confounders, and no significant association was seen. We believe that these findings support the assertion that community researchers were not significantly influenced by these factors when determining preferred diagnostic testing strategy. Given that both antigen- and PCR-based testing strategies have different strengths and intended use-cases, a preferred testing strategy depends on the specific context of community-based research.

Most projects used a PCR-based testing strategy, partially explained by early availability of PCR-based tests while antigen tests were only widely available in later phases of the pandemic. Antigen testing became more common in later phases of the pandemic, perhaps due to lower cost and convenience. However, when adjusting for the phase of the pandemic, no significant differences were seen in test choice based on race, ethnicity, or housing stability.

The earliest period of the COVID-19 pandemic was characterized by stark disparities in resources, testing, and vaccine availability throughout American society, with marginalized and minority populations particularly disadvantaged (5, 19). At the earliest points of the COVID-19 pandemic, higher rates of hospitalization and death were seen in persons from racial and ethnic minorities, which improved but did not disappear over time (20, 21).

The RADx-UP initiative intentionally targeted underserved communities to help minimize these disparities, or ameliorate disparities in access and opportunity brought to light by the pandemic. The RADx-UP consortium was able to provide an unprecedented level of resources and research opportunity to underserved and vulnerable populations. For example, the RADx-UP Community Collaboration Grant Program (C2G) enabled 70 different projects to engage with their communities, raise awareness about COVID-19, promote measures to reduce transmission and foster trust toward both public health and community-based research efforts (22).

As that research, organizational, and financial support was provided to underserved communities, understanding common themes and characteristics of support can provide key insights for future funding initiatives. We believe that the flexibility allowed to RADx-UP investigators in choosing the best testing strategy for their community was a strength, and did not result in significant differences by targeted population. An important implication of this finding is that similar large-scale funding initiatives to improve health disparities should also allow for investigator-driven preference on testing strategy. A strength of this analysis is the inclusion of a large network of diverse community-engaged COVID-19 testing projects. To our knowledge, this analysis is the first to examine the preferences of community-based health researchers on this large-scale and provide insight into the likelihood of investigators choosing different testing strategies based on the characteristics of the population they are serving. We believe understanding these preferences is an important contribution to overall assessment of community-based health research efforts throughout the COVID-19 pandemic and moving forward.

There are also noteworthy limitations of this analysis. One limitation is that it did not directly ask investigators about motivations regarding the choice of test, but instead relied on demonstrated preferences. Perhaps most likely, a principal investigator’s testing choice was most influenced by available infrastructure, budget, and preferences of test characteristics based on available information at that time. Such a hypothesis would need to be investigated further through PI interviews. However, we believe that similar to the use of discrete choice experiments in social science, examining a chosen preference remains a valid way of analyzing preference even if investigator motivation is not known directly (23). Additionally, this analysis was designed to examine if an investigator’s decision at the project level to offer antigen- versus PCR-based testing strategy was affected by the intention to target a specific population based on race, ethnicity, rurality, housing stability, or project size. However, we acknowledge the intersectional importance of all these variables on the health of an individual or population, which may or may not have been analyzable in this context. Additionally, the choice to combine projects that used PCR and antigen-based testing with PCR-only testing was done due to high rates of confirmatory testing in these projects, which was considered more similar to a PCR-only approach. Yet, this decision may have unintentionally biased our results compared to analyzing a separate “combined” category.

Additionally, the unit of analysis in this study was at the project level. Despite full analysis of projects in our consortium, there remains the possibility that meaningful differences were not detected due to a small number of projects targeting certain sociodemographic types. Specifically, only Black race had event rates sufficient for logistic regression, which limits the ability to compare across races. Differences in testing strategy at the patient level could also not be examined, and examining investigators’ choice rather than surveys of participants limits generalizability of this study. Almost all projects offered their participants only a single type of testing by a project, and testing projects that changed modalities over time were classified based on intended test type at time of project approval.

In conclusion, among community-based research projects for COVID-19 testing in RADx-UP, no significant associations were seen in investigators’ choice of rapid antigen versus PCR-based strategy and characteristics of intended population. Future community-based research efforts should continue to remain vigilant that new infectious disease diagnostic technologies are disseminated and utilized in equitable and effective ways.

## Data Availability

The original contributions presented in the study are included in the article/Supplementary material, further inquiries can be directed to the corresponding author.
