# Supplementary material for: Association of race, ethnicity, and housing stability with COVID-19 testing method by investigators in underserved populations 2020–2023
Source: Front Public Health. 2025 Sep 24;13:1605167. doi: 10.3389/fpubh.2025.1605167 (PMC12504233; doi:10.3389/fpubh.2025.1605167)
Supplement: Supplementary file 1 [file Table_1.DOCX]

**Supplementary material:**

Variable definitions:

Exposures for race, ethnicity, rurality and unstable housing were determined using keywords searches in the Project Structure Metadata and Study Design survey “Primary Target Population” and “Secondary Target Population” columns. See Table S.1 for key word definitions. If a keyword was not found, it is assumed that the project did not intend to study that exposure group of interest. Region was a complete variable based on mapping the Geographic Location of the study listed in the Project Structure Metadata and Study Design survey to its region in the United States. Projects conducted in more than one region were assigned a value of “multiple regions”. Phase of Pandemic was derived from the date the testing project was greenlit by the RADx-UP Testing Core. All variables were complete. Variable sources are listed in Table S.2.

Reference categories for each model was “did not intend <characteristic> as a target population”.

| **Table S.1:** Exposure and Adjustment Variable Key Words | |
| --- | --- |
| **Variable** | **Key Word(s)** |
| Race |  |
| Black | Black, African American |
| Asian, Native Hawaiian, Pacific Islander | Asian, Hawaiian, Pacific, OPI |
| American Indian/Alaskan Native | American Indian, Alaskan |
|  |  |
| Ethnicity | Hispanic, Latinx |
| Unstable Housing | Low income, housing, homeless |
| Rurality | Rural, Non-rural |

| **Table S.2:** Specific Variable Sources | |
| --- | --- |
| **Data Source** | **Variable** |
| RADx-UP Project Structure Metadata and Study Design | Race |
|  | Ethnicity |
|  | Unstable Housing |
|  | Rurality |
|  | Region |
| Core Analytic Datasets – RADx-UP CDCC | Project size |
| RADx-UP Testing Core Green Light Data | Phase of Pandemic  Intended Test Type |

Supplementary Figure 1: CONSORT Diagram


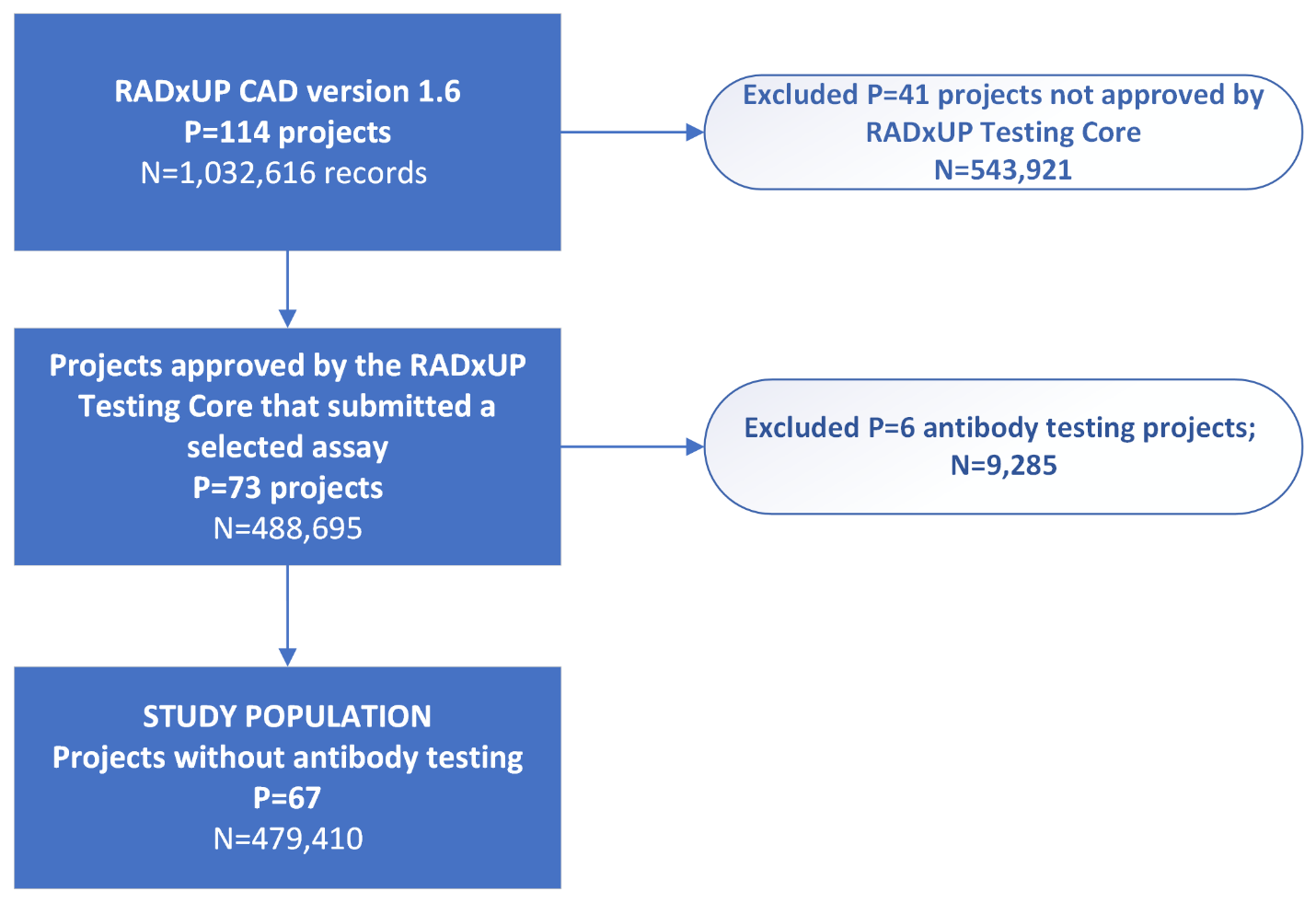


Tables S.3A-S.3D display the participant-level demographics Overall and by the Target Population of the project. These tables show us that the Observed characteristics line up with the Intended Target Population. For example, Table S.2A shows that projects whose intended target population included Black participants had higher percentages of Black participants in the Observed population compared to projects that did not indicate Black participants were part of their Primary Target Population. This pattern repeats for the other main exposures in Tables S.2B and S.2D. However, the pattern does not hold when comparing projects who identified AI/AN individuals in their Primary Target Population. There is a higher proportion of AI/AN individuals in the projects that did not include this group in their Primary Target Population. However, there were very few participants among the projects that did identify AI/AN individuals in their Primary Target Population.

**Table S.3A: Comparing Demographics in Observed Population^0^ by Primary Target Population**

| **Characteristic** | **Overall (N=67)** | **Black (N=21)** | **Not Black (N=46)** |
| --- | --- | --- | --- |
| Race |  |  |  |
| American Indian or Alaska Native | 3721/479410 (0.8%) | 127/30071 (0.4%) | 3594/449339 (0.8%) |
| Black or African American | 34067/479410 (7.1%) | 8143/30071 (27.1%) | 25924/449339 (5.8%) |
| Asian | 4128/479410 (0.9%) | 131/30071 (0.4%) | 3997/449339 (0.9%) |
| Native Hawaiian or Other Pacific Islander | 6611/479410 (1.4%) | 13/30071 (0.0%) | 6598/449339 (1.5%) |
| White | 249967/479410 (52.1%) | 2749/30071 (9.1%) | 247218/449339 (55.0%) |
| Some other race | 66778/479410 (13.9%) | 697/30071 (2.3%) | 66081/449339 (14.7%) |
| Multiple race | 5790/479410 (1.2%) | 322/30071 (1.1%) | 5468/449339 (1.2%) |
| Prefer not to answer | 25819/479410 (5.4%) | 288/30071 (1.0%) | 25531/449339 (5.7%) |
| missing | 82529/479410 (17.2%) | 17601/30071 (58.5%) | 64928/449339 (14.4%) |
| Hispanic |  |  |  |
| Yes, of Hispanic, Latino, or Spanish origin | 103781/479410 (21.6%) | 2764/30071 (9.2%) | 101017/449339 (22.5%) |
| No, not of Hispanic, Latino or Spanish origin | 139151/479410 (29.0%) | 8205/30071 (27.3%) | 130946/449339 (29.1%) |
| Prefer not to answer | 16923/479410 (3.5%) | 94/30071 (0.3%) | 16829/449339 (3.7%) |
| missing | 219555/479410 (45.8%) | 19008/30071 (63.2%) | 200547/449339 (44.6%) |
| ^0^the Observed Population represents participant level records from the RADxUP Core Analytic Datasets v1.6 | | | |

**Table S.3B: Comparing Demographics in Observed Population^0^ by Primary Target Population**

| **Characteristic** | **Overall (N=67)** | **Asian/NH/OPI (N=7)** | **Not Asian/NH/OPI (N=60)** |
| --- | --- | --- | --- |
| Race |  |  |  |
| American Indian or Alaska Native | 3721/479410 (0.8%) | 39/15332 (0.3%) | 3682/464078 (0.8%) |
| Black or African American | 34067/479410 (7.1%) | 277/15332 (1.8%) | 33790/464078 (7.3%) |
| Asian | 4128/479410 (0.9%) | 621/15332 (4.1%) | 3507/464078 (0.8%) |
| Native Hawaiian or Other Pacific Islander | 6611/479410 (1.4%) | 4726/15332 (30.8%) | 1885/464078 (0.4%) |
| White | 249967/479410 (52.1%) | 1542/15332 (10.1%) | 248425/464078 (53.5%) |
| Some other race | 66778/479410 (13.9%) | 283/15332 (1.8%) | 66495/464078 (14.3%) |
| Multiple race | 5790/479410 (1.2%) | 2744/15332 (17.9%) | 3046/464078 (0.7%) |
| Prefer not to answer | 25819/479410 (5.4%) | 48/15332 (0.3%) | 25771/464078 (5.6%) |
| missing | 82529/479410 (17.2%) | 5052/15332 (33.0%) | 77477/464078 (16.7%) |
| Hispanic |  |  |  |
| Yes, of Hispanic, Latino, or Spanish origin | 103781/479410 (21.6%) | 917/15332 (6.0%) | 102864/464078 (22.2%) |
| No, not of Hispanic, Latino or Spanish origin | 139151/479410 (29.0%) | 5029/15332 (32.8%) | 134122/464078 (28.9%) |
| Prefer not to answer | 16923/479410 (3.5%) | 102/15332 (0.7%) | 16821/464078 (3.6%) |
| missing | 219555/479410 (45.8%) | 9284/15332 (60.6%) | 210271/464078 (45.3%) |
| ^0^the Observed Population represents participant level records from the RADxUP Core Analytic Datasets v1.6 | | | |

**Table S.3C: Comparing Demographics in Observed Population^0^ by Primary Target Population**

| **Characteristic** | **Overall (N=67)** | **Alaskan Native/American Indian (N=5)** | **Not Alaskan Native/American Indian (N=62)** |
| --- | --- | --- | --- |
| Race |  |  |  |
| American Indian or Alaska Native | 3721/479410 (0.8%) | 5/1082 (0.5%) | 3716/478328 (0.8%) |
| Black or African American | 34067/479410 (7.1%) | 47/1082 (4.3%) | 34020/478328 (7.1%) |
| Asian | 4128/479410 (0.9%) | 31/1082 (2.9%) | 4097/478328 (0.9%) |
| Native Hawaiian or Other Pacific Islander | 6611/479410 (1.4%) | 1/1082 (0.1%) | 6610/478328 (1.4%) |
| White | 249967/479410 (52.1%) | 364/1082 (33.6%) | 249603/478328 (52.2%) |
| Some other race | 66778/479410 (13.9%) | 49/1082 (4.5%) | 66729/478328 (14.0%) |
| Multiple race | 5790/479410 (1.2%) | 7/1082 (0.6%) | 5783/478328 (1.2%) |
| Prefer not to answer | 25819/479410 (5.4%) | 43/1082 (4.0%) | 25776/478328 (5.4%) |
| missing | 82529/479410 (17.2%) | 535/1082 (49.4%) | 81994/478328 (17.1%) |
| Hispanic |  |  |  |
| Yes, of Hispanic, Latino, or Spanish origin | 103781/479410 (21.6%) | 768/1082 (71.0%) | 103013/478328 (21.5%) |
| No, not of Hispanic, Latino or Spanish origin | 139151/479410 (29.0%) | 121/1082 (11.2%) | 139030/478328 (29.1%) |
| Prefer not to answer | 16923/479410 (3.5%) | 11/1082 (1.0%) | 16912/478328 (3.5%) |
| missing | 219555/479410 (45.8%) | 182/1082 (16.8%) | 219373/478328 (45.9%) |
| ^0^the Observed Population represents participant level records from the RADxUP Core Analytic Datasets v1.6 | | | |

**Table S.3D: Comparing Demographics in Observed Population^0^ by Primary Target Population**

| **Characteristic** | **Overall (N=67)** | **Hispanic (N=33)** | **Not Hispanic (N=34)** |
| --- | --- | --- | --- |
| Race |  |  |  |
| American Indian or Alaska Native | 3721/479410 (0.8%) | 2977/227845 (1.3%) | 744/251565 (0.3%) |
| Black or African American | 34067/479410 (7.1%) | 7462/227845 (3.3%) | 26605/251565 (10.6%) |
| Asian | 4128/479410 (0.9%) | 2341/227845 (1.0%) | 1787/251565 (0.7%) |
| Native Hawaiian or Other Pacific Islander | 6611/479410 (1.4%) | 1662/227845 (0.7%) | 4949/251565 (2.0%) |
| White | 249967/479410 (52.1%) | 127757/227845 (56.1%) | 122210/251565 (48.6%) |
| Some other race | 66778/479410 (13.9%) | 33167/227845 (14.6%) | 33611/251565 (13.4%) |
| Multiple race | 5790/479410 (1.2%) | 2128/227845 (0.9%) | 3662/251565 (1.5%) |
| Prefer not to answer | 25819/479410 (5.4%) | 24895/227845 (10.9%) | 924/251565 (0.4%) |
| missing | 82529/479410 (17.2%) | 25456/227845 (11.2%) | 57073/251565 (22.7%) |
| Hispanic |  |  |  |
| Yes, of Hispanic, Latino, or Spanish origin | 103781/479410 (21.6%) | 96184/227845 (42.2%) | 7597/251565 (3.0%) |
| No, not of Hispanic, Latino or Spanish origin | 139151/479410 (29.0%) | 93795/227845 (41.2%) | 45356/251565 (18.0%) |
| Prefer not to answer | 16923/479410 (3.5%) | 16459/227845 (7.2%) | 464/251565 (0.2%) |
| missing | 219555/479410 (45.8%) | 21407/227845 (9.4%) | 198148/251565 (78.8%) |
| ^0^the Observed Population represents participant level records from the RADxUP Core Analytic Datasets v1.6 | | | |

Table S.4 compares the demographics in the Observed Population Overall and by Intended Test Type. There are a higher proportion of Black participants in the projects that selected test types that Include PCR compared to projects that only selected Antigen tests. There is a very high degree of missingness on the Hispanic ethnicity variable among projects that selected test types that Include PCR, and a much higher percentage of Hispanic participants among projects that selected Antigen only testing. More projects in the West selected Antigen only testing while more projects in the South opted for Inclusion of PCR. In general, projects that selected Antigen only testing had a larger number of participants.

**Table S.4: Comparing Demographics in Observed Population^0^ by Intended Test Type**

| **Characteristic** | **Overall (N=67)** | **Antigen Only (N=24)** | **Inclusion of PCR (N=43)** |
| --- | --- | --- | --- |
| Race |  |  |  |
| American Indian or Alaska Native | 3721/479410 (0.8%) | 2635/230039 (1.1%) | 1086/249371 (0.4%) |
| Black or African American | 34067/479410 (7.1%) | 4224/230039 (1.8%) | 29843/249371 (12.0%) |
| Asian | 4128/479410 (0.9%) | 2537/230039 (1.1%) | 1591/249371 (0.6%) |
| Native Hawaiian or Other Pacific Islander | 6611/479410 (1.4%) | 2649/230039 (1.2%) | 3962/249371 (1.6%) |
| White | 249967/479410 (52.1%) | 119909/230039 (52.1%) | 130058/249371 (52.2%) |
| Some other race | 66778/479410 (13.9%) | 27900/230039 (12.1%) | 38878/249371 (15.6%) |
| Multiple race | 5790/479410 (1.2%) | 4029/230039 (1.8%) | 1761/249371 (0.7%) |
| Prefer not to answer | 25819/479410 (5.4%) | 22716/230039 (9.9%) | 3103/249371 (1.2%) |
| missing | 82529/479410 (17.2%) | 43440/230039 (18.9%) | 39089/249371 (15.7%) |
| Hispanic |  |  |  |
| Yes, of Hispanic, Latino, or Spanish origin | 103781/479410 (21.6%) | 80824/230039 (35.1%) | 22957/249371 (9.2%) |
| No, not of Hispanic, Latino or Spanish origin | 139151/479410 (29.0%) | 116621/230039 (50.7%) | 22530/249371 (9.0%) |
| Prefer not to answer | 16923/479410 (3.5%) | 16154/230039 (7.0%) | 769/249371 (0.3%) |
| missing | 219555/479410 (45.8%) | 16440/230039 (7.1%) | 203115/249371 (81.5%) |
| Rural |  |  |  |
| Urban | 112606/479410 (23.5%) | 18440/230039 (8.0%) | 94166/249371 (37.8%) |
| Rural | 31125/479410 (6.5%) | 978/230039 (0.4%) | 30147/249371 (12.1%) |
| missing | 335679/479410 (70.0%) | 210621/230039 (91.6%) | 125058/249371 (50.1%) |
| Region |  |  |  |
| Midwest | 14455/479410 (3.0%) | 1494/230039 (0.6%) | 12961/249371 (5.2%) |
| Northeast | 4484/479410 (0.9%) | 1805/230039 (0.8%) | 2679/249371 (1.1%) |
| Other US territory | 3829/479410 (0.8%) | 0/230039 (0.0%) | 3829/249371 (1.5%) |
| South | 192739/479410 (40.2%) | 3639/230039 (1.6%) | 189100/249371 (75.8%) |
| West | 210773/479410 (44.0%) | 187683/230039 (81.6%) | 23090/249371 (9.3%) |
| missing | 53130/479410 (11.1%) | 35418/230039 (15.4%) | 17712/249371 (7.1%) |
| Project size |  |  |  |
| Mean (SD) | 107635 (68453) | 123709 (65174) | 92807 (68057) |
| Median (Q1, Q3) | 148641 (23483, 164513) | 164513 (35363, 164513) | 148641 (16531, 148641) |
| Min, Max | 32, 164513 | 95, 164513 | 32, 148641 |
| missing | 0 | 0 | 0 |
| ^0^the Observed Population represents participant level records from the RADxUP Core Analytic Datasets v1.6 | | | |

**Table S.5:** Project-specific Study Design Metadata

| **Project*** | **Geographic location** | **Testing vs non-testing projects** | **Target population (primary)** | **Target population (secondary)** | **Study design** | **Study setting** | **Mode of data collection** | **Study data collection start date** | **Vaccine availability phase*** |
| --- | --- | --- | --- | --- | --- | --- | --- | --- | --- |
| 10 | Maryland, Missouri | Testing | Intellectual and Developmental Disabilities | -- | Cluster randomized trial | Schools | Questionnaire/survey (in-person)Questionnaire/survey(online or by mail)COVID-19 testing (choose this respones if your project is administering COVID 19 testing) | 11/19/2020 | Pre-vaccine (before 12/14/20) |
| 105 | CaliforniaNew MexicoTexas | Testing | African AmericanHispanic/LatinXAmerican IndiansAsianHawaiian/ Pacific IslandersOlder AdultsLow income populations | African AmericanHispanic/LatinXAmerican IndiansAsianHawaiian/ Pacific IslandersOlder Adults | Clustered randomized trial | Other - dialysis facilities | Questionnaire/survey (in-person)COVID-19 testing (choose this response if your project is administering COVID 19 testing)Electronic Health Records | 02/06/2023 | Post Phase 4 - Vaccine available to all (after 05/23/22) |
| 106 | Illinois | Testing | African American | -- | Randomized controlled trial | Community health center | Questionnaire/survey (online or by mail) | 04/04/2022 | Phase 4 - First booster available to all (between 11/22/21 and 05/23/22) |
| 12 | New York | Testing | Low income housing | None | Cross-sectional cohortRandomized controlled trial | Home [e.g. mailed and email surveys, in-home interviews] | Virtual focus group text message surveyCovid -19 tests data from EHR/ Secondary medical records; Electronic Health Records | 03/08/2021 | Phase 1 - First vaccine delivered and available to certain populations (between 12/14/20 and 04/19/21 |
| 14 | Maryland | Testing | Hispanic / LatinXImmigrants | -- | Case reports | Other hotline callsCommunity health centerCommunity centers | Electronic Health Records | 10/01/2020 | Pre-vaccine (before 12/14/20) |
| 18 | Florida | Testing | African American Hispanic / LatinXOlder AdultsAdults with substance useLow income populations Low income housing People Experiencing HomelessnessFederally Qualified Health Centers (FQHCs) People living with HIV/AIDS (PLWHA) | -- | Cross sectional cohort | Community centersOther community (non-clincial) settings - In collaboration with community partners we tested participants at schools, churches, community centersMobile testing unitCommunity health center | InterviewQuestionnaire/survey (online or by mail)COVID-19 testing Other biological data (e.g. blood sample, nasal swab) Electronic Health RecordsCOVID-19 tests data from Electronic Health Records/Secondary Medical Records | 05/18/2021 | Phase 2 - First vaccine available to all (between 04/20/21 and 09/25/21) |
| 2 | Alabama | Testing | Rural Populations | -- | Cross-sectional cohort | Home (e.g. mailed and email surveys, in-home interviews) | Interview Questionnaire/survey (online or by mail) | 09/23/2020 | Pre-vaccine (before 12/14/20) |
| 23 | West Virginia | Testing | Rural Populations | African American | Cluster randomized trialCross-sectional cohortOther not randomized | Mobile testing unitHome (e.g. mailed and email surveys, in-home interviews) | COVID-19 testing InterviewQuestionnaire/survey (in person) | 12/01/2020 | Pre-vaccine (before 12/14/20) |
| 25 | Arizona | Testing | African AmericanHispanic/LatinXAmerican IndiansAlaskan NativeTesting Deserts | AsianHawaiian / Pacific IslandersOlder AdultsRural PopulationsFarm WorkersLow income populations | Prospective observational cohort study | SchoolsCommunity centersGroup homes (e.g.halfway house, nursing homes)Community health center | Questionnaire/survey (in-person)Questionnaire/survey (online or by mail)COVID-19 testing (choose this response if your project is administering COVID19 testing) | 02/27/2021 | Phase 1 - First vaccine delivered and available to certain populations (between 12/14/20 and 04/19/21 |
| 26 | Hawaii | Testing | Hawaiian / Pacific IslandersLow income population | -- | Cross-sectional cohort | SchoolsCommunity centersCommunity health center | Questionnaire/survey (in-person)Questionnaire/survey (onelien or by mail)COVID-19 testing (choose this response if your project is administering COVID 19 testing) | 03/29/2021 | Phase 1 - First vaccine delivered and available to certain populations (between 12/14/20 and 04/19/21 |
| 27 | US Virgin Islands/Puerto Rico | Testing | African AmericanHispanic / LatinXFederally Qualified Health Centers (FQHCs) | ImmigrantsLGBTQ/Sexual and Gender Minority (SGM)Rural populationsLow income populationsLow income housing | Prospective observational cohort study | Community centersOutpatient clinicMobile testing unitCommunity health center | Questionnaire/survey (in-person)Questionnaire/survey (online or by mail)Covid-19 TestingOther biological data (e.g. blood sample, nasal swab) | 09/22/2021 | Phase 2 - First vaccine available to all (between 04/20/21 and 09/25/21) |
| 3 | Maryland | Testing | African AmericanHispanic / LatinX | -- | Randomized controlled trial | Home (e.g. mailed and email surveys, in-home interviews)Outpatient clinicMobile testing unit | Interview Questionnaire/survey (online or by mail)COVID-19 testingOther biological data (e.g. blood sample, nasal swab) | 02/17/2021 | Phase 1 - First vaccine delivered and available to certain populations (between 12/14/20 and 04/19/21 |
| 30 | Arkansas IllinoisIndianaLouisianaTexas | Testing | People with a history of exposure to law enforcementHispanic/Latin XLow income populationsOther non-incarcerated people with a history of criminal justice involvement | African AmericanLGBTQ/Sexual and Gender Minority (SGM)African AmericanAdults with substance useYouth | Randomized controlled trial | Other community (non-clinical)setting - community - based organizationOutpatient clinicCommunity health center | Questionnaire/survey (in-person)Questionnaire/survey (online or by mail)COVID-19 testing (choose thie response if your project is administering COVID 19 testing) | 04/29/2021 | Phase 2 - First vaccine available to all (between 04/20/21 and 09/25/21) |
| 31 | FloridaMinnesotaRhode IslandWashington | Non-testing | Incarcerated people | African AmericanHispanic/LatinXAmerican IndiansAdults with substance useRural PopulationsLow income populationsPeople Experiencing HomelessnessPeople living with HIV/AIDS (PLWHA) | Retrospective data | No participant contact (e.g. EHR studies) | Electronic Health Records | 03/22/2021 | Phase 1 - First vaccine delivered and available to certain populations (between 12/14/20 and 04/19/21 |
| 35 | Utah | Testing | Hispanic/LatinXRural populationsLow income populationsFederally Qualified Health Centers (FQHCs) | African AmericanHispanic/Latin XAmerican IndiansAsianHawaiian/Pacific IslandersImmigrantsTesting Deserts | Randomized controlled trial | Community health center | Eletronic Health Records | 02/23/2021 | Phase 1 - First vaccine delivered and available to certain populations (between 12/14/20 and 04/19/21 |
| 36 | Ohio | Testing | Hispanic/LatinX | -- | Cross-sectional cohort | Community health centerHome (e.g. mailed and email surveys, in-home interviews)Community centerOther community (non-clinical) center | Questionnaire/survey (online or by mail) | 08/03/2021 | Phase 2 - First vaccine available to all (between 04/20/21 and 09/25/21) |
| 37 | New Jersey | Testing | African AmericanHispanic / Latin XOther healthcare workers | Low income populations | Cross-sectional cohort | Community health center | InterviewQuestionnaire/survey (online or by mail)Qualitative Focus GroupCOVID-19 testing (choose this response if your project is administering COVID 19 testing) | 11/19/2020 | Pre-vaccine (before 12/14/20) |
| 38 | Texas | Testing | African AmericanHispanic / LatinXAsianRural PopulationsLow income populations | ImmigrantsOlder AdultsPregnant WomenLow income housing | Cross-sectional cohort | Home (e.g. mailed and email surveys, in-home interviews) | Questionnaire/survey [online or by mail] | 03/14/2022 | Phase 4 - First booster available to all (between 11/22/21 and 05/23/22) |
| 4 | New York | Testing | Low income housing | -- | Randomized controlled trialCluster randomzied trial | Home (e.g. mailed and emamil surveys, in-home inerviews) | InterviewCOVID-19 testing (choose this response if your projecct is administered COVID 19 testing)Qualitative Focus Group | 08/02/2021 | Phase 2 - First vaccine available to all (between 04/20/21 and 09/25/21) |
| 40 | California | Testing | Hispanic/LatinX | ImmigrantsRural PopulationsFarm WorkersLow income housing | Cross-sectional cohort | Mobile testing unitCommunity health centerOther-Mass testing event in outdoor public settingSchoolsOther community (non-clinical) settings-outdoor pop-up testing and testing for employees at their place of business | Questionnaire/survey (in-person)COVID-19 testing (choose this response if your project is administering COVID 19 testing) | 01/28/2021 | Phase 1 - First vaccine delivered and available to certain populations (between 12/14/20 and 04/19/21 |
| 41 | Montana, Washington | Testing | Hispanic/LatinXAmerican Indians | -- | Cross-sectional cohortRandomized controlled trial | Home (e.g. mailed and email surveys, in-home interviews) | Questionnaire/survey (in-person)Questionnaire/survey (online or by mail)COVID-19 testing (choose this response if your project is administering COVID 19 testing) | 04/26/2021 | Phase 2 - First vaccine available to all (between 04/20/21 and 09/25/21) |
| 42 | Illinois | Testing | Federally Qualified Health Centers (FQHCs) | Testing Deserts | Prospective observational cohort | Outpatient clinicHospitalOther community (non-clinical) settings (specify) - urban communities in Cook and suburban Cook counties of Illinois | COVID-19 testingQuestionnaire/survey (in-person)Questionnaire/survey (online or by mail) | 04/19/2021 | Phase 1 - First vaccine delivered and available to certain populations (between 12/14/20 and 04/19/21 |
| 44 | Missouri | Testing | African American | -- | Cluster randomized trial | Other-church | Questionnaire/survey (in-person)Questionnaire/survey (online or by mail)COVID-19 testing (choose this response if your project is administering COVID 19 testing) | 04/11/2021 | Phase 1 - First vaccine delivered and available to certain populations (between 12/14/20 and 04/19/21 |
| 46 | Puerto Rico | Testing | Hispanic/LatinX | -- | Cross-sectional cohort | Community centersMobile testing unit | InterviewQuestionnaire/survey (in-person) | 03/22/2021 | Phase 1 - First vaccine delivered and available to certain populations (between 12/14/20 and 04/19/21 |
| 47 | New York | Testing | Adults with substance use | Low income populations | Cross-sectional cohortProspective Observational Cohort | Other community (non-clinical) settings (specify): Two areas in New York City, including northern Manhattan (Washington Heights and Harlem) and the South Bronx, areas served by two community based organizations (CBOs) specializing in substance use and substance use disorders (SUD) treatment and providing support services for individuals who abuse substances: Argus Community Inc., in Bronx, NY and Alliance for Positive Change, New York, NY. | InterviewQuestionnaire/survey (in-person)Questionnaire/survey (online or by mail)Qualitative Focus GroupCOVID-19 testingOther biological data (nasal swab)Electronic Health Records | 06/07/2021 | Phase 2 - First vaccine available to all (between 04/20/21 and 09/25/21) |
| 48 | North Carolina | Testing | Hispanic / LatinXAfrican AmericanRural Populations | Farm workers | Case reports | Mobile testing units | Interview (zoom)Qualitative Focus GroupQuestionnaire | 10/21/2021 | Phase 3 - First booster available to certain populations (between 09/26/21 and 11/21/21) |
| 49 | Alabama | Testing | African American | Low income populations | Prospective observational cohort studyCross-sectional cohort | Mobile testing unit | Questionnaire/survey (in-person)COVID-19 testing (choose this response if your project is adminstering COVID 19 testing) | 06/16/2021 | Phase 2 - First vaccine available to all (between 04/20/21 and 09/25/21) |
| 50 | Pennsylvania | Testing | African AmericanHispanic / Latin XLow income populationsLow income housingPeople Experiencing HomelessnessFederally Qualified Health Centers (FQHCs)Testing Desers | -- | Prospective observational cohort studyRandomized controlled trial | Home (e.g.mailed and email surveys, in-home interviews)Outpatient clinicCommunity health center | InterviewQuestionnaire/survey (in-person)Questionnaire/survey (online or by mail) | 05/20/2021 | Phase 2 - First vaccine available to all (between 04/20/21 and 09/25/21) |
| 51 | Texas | Testing | Hispanic/LatinX | Hispanic/LatinXRural populations | Cross-sectional cohort | SchoolsMobile testing unitOther-School [University of Texas El Paso] SVFHC Food pantries Mobile testing unit | Questionnaire/survey (online or by mail)COVID-19 testing (choose this response if your project is administering COVID 19 testing) | 12/14/2021 | Phase 4 - First booster available to all (between 11/22/21 and 05/23/22) |
| 52 | Louisiana | Testing | Low-income populations, African American | -- | Cross-sectional cohort | SchoolsCommunity centersOther community (non-clinical) settings-ChurchesOutpatient clinicMobile testing unit from in or near 5 zip codesHome (e.g. mailed and email surveys, in-home interviews) | Questionnaire/survey (in-person)COVID-19 testing (choose this response if your project is administering COVID 19 testing)Qualitative Focus Group | 02/10/2021 | Phase 1 - First vaccine delivered and available to certain populations (between 12/14/20 and 04/19/21 |
| 55 | Guam, Hawaii | Testing | Hawaiian / Pacific IslandersNon-native Hawaiian / Pacific Islanders | Hawaiian / Pacific IslandersNon-native Hawaiian / Pacific Islanders | Prospective observational cohort study | Other community (non-clinical) settings (specify)Public housingChurches, and through networks of project partners | Questionnaire/survey (online or by mail)COVID-19 testingVirtual Focus Group | 05/21/2021 | Phase 2 - First vaccine available to all (between 04/20/21 and 09/25/21) |
| 56 | Illinois | Testing | African American | -- | Cross-sectional cohort | Other-At home COVID 19 testingOther community (non-clinical) setting-churchesFood pantrieschurch based eventscampssports practices and games | Questionnaire/survey (in-person)Questionnaire/survey (oneline or by mail)COVID-19 testing (choose this response if your project is administering COVID 19 testing) | 03/25/2021 | Phase 1 - First vaccine delivered and available to certain populations (between 12/14/20 and 04/19/21 |
| 57 | California | Testing | Hispanic/LatinX | -- | Cross-sectional cohort | Other community (non-clinical) setting - Community vaccine events, Health and Wellness Fair and school events | Questionnaire/survey (in-person)COVID-19 testing (choose this response if your project is administering COIVD 19 testing)Electronic Health Records | 06/25/2022 | Post Phase 4 - Vaccine available to all (after 05/23/22) |
| 58 | Arkansas | Testing | Hispanic/LatinXHawaiian / Pacific Islanders | -- | Cross-sectional cohort | Community centersOther community (non-clinical) settings- Community events (cultural events, holiday celebrations etc.)Mobile testing unit | Questionnaire/survey (in-person)COVID-19 testing (choose this response if your project is administering COVID 19 testing)COVID -19 test data from Electronic Health Records/Secondary Medical Records | 12/08/2020 | Pre-vaccine (before 12/14/20) |
| 6 | California | Testing | Hispanic / LatinX | None | Prospective observational cohort | Mobile testing unit | Interview | 02/03/2022 | Phase 4 - First booster available to all (between 11/22/21 and 05/23/22) |
| 64 | Maryland | Testing | Adults with substance use [WWOD women who use drugs] | Sex workersWomen Experiencing Homelessness | Case report [Free listing, Pile sorting] Case report [Ethnographic observations] Prospective observational cohort study [Multi-method longitudinal study] | Community centersNon-clinical community (drop-in centers, community organizations, streets)Community health center and outpatient clinicMobile testing unitOther community non-clinical setting (streets where drug use is common) | InterviewsQuestionnaire/survey (in-person)COVID-19 test data from Electronic Health Records / Secondary Medical RecordsObservations for all aims | 08/18/2021 | Phase 2 - First vaccine available to all (between 04/20/21 and 09/25/21) |
| 65 | New Mexico, Wyoming | Testing | Coal miners | American Indian and Hispanic | Cross-sectionalProspective observational cohort study | Other- coal mines | COVID-19 testing (choose this response if your project is administering COVID 19 testing) | 02/01/2021 | Phase 1 - First vaccine delivered and available to certain populations (between 12/14/20 and 04/19/21 |
| 67 | Oregon | Testing | Adults with substance use | -- | Prospective observational cohort study | Other-HIV Alliance’s (HIVA) Syringe Service Centers | COVID-19 testing [choose this response if your project is administering COVID 19 testing] | 03/03/2021 | Phase 1 - First vaccine delivered and available to certain populations (between 12/14/20 and 04/19/21 |
| 7 | California | Testing | Hispanic/LatinXPregnant women | African AmericanAsianImmigrantsOlder AdultsLow income populationsLow income housingPeople Experiencing HomelessnessFederally Qualified Health Centers (FQHCs) | Cross-sectional cohort | Community health center | Questionnaire/survey (in-person)Questinnaire/survey (online or by mail)COVID-19 testing (choose this response if your project is administering COVID-19 testing) | 12/29/2020 | Phase 1 - First vaccine delivered and available to certain populations (between 12/14/20 and 04/19/21 |
| 70 | New Jersey | Testing | Low income populations | African AmericanHispanic / LatinXLow income housingPeople Experiencing HomelessnessPeople living with HIV/AIDS (PLWHA) | Randomized controlled trial | Home (e.g. mailed and email surveys, in-homeinterviews) | InterviewQuestionnaire/survey (online or by mail)Qualitative Focus Group | 02/12/2021 | Phase 1 - First vaccine delivered and available to certain populations (between 12/14/20 and 04/19/21 |
| 71 | North Carolina | Testing | Other-students K-12 grade and staff | -- | Prospective observational cohort study | Schools | Questionnaire/survey (online or by mail)COVID-19 testing (choose this response if your project is administering COVID 19 testing) | 04/30/2021 | Phase 2 - First vaccine available to all (between 04/20/21 and 09/25/21) |
| 72 | Illinois, Missouri | Testing | African AmericanLow income populationsOther-African American students (k-12), their families and staff | -- | Prospective observational cohort studyCluster randomized trial | SchoolsOther-Home COVID-19 testing provided | Questionnaire/survey (in-person)Questionnaire/survey (online or by mail)COVID-19 testing (choose this response if your project is administering COVID 19 testing) | 05/14/2021 | Phase 2 - First vaccine available to all (between 04/20/21 and 09/25/21) |
| 73 | New York | Testing | Intellectual and Developmental Disabilities | Women Experiencing Homelessness | Prospective observational Cohort | Schools | InterviewQuestionnaire/survey (in-person)Questionnaire/survey (online or by mail)COVID-19 testing (choose this response if your project is administering COVID 19 testing) | 05/11/2021 | Phase 2 - First vaccine available to all (between 04/20/21 and 09/25/21) |
| 74 | Wisconsin | Testing | Other-children with special needs | -- | Prospective observational cohort study | Home (e.g. mailed and email surveys, in-home interviews)Schools | Questionnaire/survey (online or by mail)COVID-19 testing (choose this response if your project is administering COVID-19 testing) | 05/17/2021 | Phase 2 - First vaccine available to all (between 04/20/21 and 09/25/21) |
| 76 | Missouri | Testing | Other-students and staff from Kansas city public schools and individuals who "walk-up" for testing at the community based site | -- | Prospective observational cohort study | SchoolsOther-Home COVID-19 testingMobile testing unitOther-Community center for COVID-19 testing | Questionnaire/survey (online or by mail)COVID-19 testing (choose this response if your project is administering COVID 19 testing) | 05/10/2021 | Phase 2 - First vaccine available to all (between 04/20/21 and 09/25/21) |
| 78 | District of ColumbiaMarylandMissouriPennsylvaniaVirginia | Testing | Intellectual and Developmental Disabilities | African American | Prospective observational cohort study | Schools | COVID-19 testing (choose this response if your project is administering COVID 19 testing) | 06/28/2021 | Phase 2 - First vaccine available to all (between 04/20/21 and 09/25/21) |
| 80 | Arizona | Testing | Hispanic/Latin XLower income population | -- | Cross sectional cohortCluster randomized trial | Schools | Questionnaire/survey (in-person)Questionnaire/survey (online or by mail)Covid-19 testing (choose this response if your project is administering COVID 19 testing) | 11/30/2021 | Phase 4 - First booster available to all (between 11/22/21 and 05/23/22) |
| 81 | Hawaii | Testing | Hawaiian/ Pacific Islanders | Low income populations | Cross-sectional cohort | Schools | Questionnaire/survey (in-person)Questionnaire/survey (online or by mail)COVID-19 testing (choose this response if your project is administering COVID 19 testing) | 07/16/2021 | Phase 2 - First vaccine available to all (between 04/20/21 and 09/25/21) |
| 83 | Nebraska | Testing | Farm Workers | Hispanic/LatinXImmigrantsRural populationsLow income populationsLow income housingTesting Deserts | Prospective observational cohort study | Home (e.g. mailed and email surveys, in-home interviews) | InterviewQuestionnaire/survey (in-person)Questionnaire/survey (online or by mail)Qualitative Focus GroupCOVID-19 testing (choose this response if your project is administering COVID 19 testing) | 03/04/2022 | Phase 4 - First booster available to all (between 11/22/21 and 05/23/22) |
| 84 | California | Testing | Hispanic / LatinXLow income populations | -- | Prospective observational cohort study | SchoolsOther home COVID -19 testing | COVID-19 testing (choose this response if your project is adminstering COVID 19 testing)COVID-19 tests data from Electronic Health Records / Secondary medical RecordsQuestionnaire/survey (by phone) | 10/18/2021 | Phase 3 - First booster available to certain populations (between 09/26/21 and 11/21/21) |
| 85 | Utah | Testing | -- | -- | Randomized controlled trial | Home (e.g. mailed and email surveys, in-home interviews) | Questionnaire/survey (online or by mail) | 01/21/2022 | Phase 4 - First booster available to all (between 11/22/21 and 05/23/22) |
| 87 | Florida | Testing | African AmericanHispanic/LatinXImmigrantsOlder AdultsLBGTQ/Sexual and Gender Minority (SGM)Adults with substance useLow income populationsLow income housingPeople Experiencing HomelessnessPeople living with HIV/AIDS (PLWHA) | -- | Prospective observational cohort studyCross-sectional cohort | Community centersOther community (non-clinical) settings-SchoolOther community (non-clinical) settings-ChurchOutpatient clinicMobile testing unitCommunity health center | InterviewQuestionnaire/survey (in-person)COVID-19 testing (choose this response if your project is administering COVID 19 testing)Other biological data (e.g. blood sample, nasal swab)Electronic Health Records | 05/24/2022 | Post Phase 4 - Vaccine available to all (after 05/23/22) |
| 88 | Puerto Rico | Testing | Hispanic / LatinXOlder Adults | LGBTQ/Sexual and Gender Minority (SGM)Rural PopulationsLow income populationsOther-Homeless | Prospective observational cohort studyCross-sectional cohort | Home (e.g. mailed and email surveys, in-home interviews)Community centersGroup homes (e.g. halfway house, nursing homes)Community health center | InterviewCOVID-19 testing (choose this response if your project is administering COVID 19 testing) | 12/14/2022 | Post Phase 4 - Vaccine available to all (after 05/23/22) |
| 9 | Oregon | Testing | Hispanic/ LatinXImmigrants | Hispanic/Immigrant | Randomized controlled trial | Home [e.g.mailed and email surveys, in-home interviews]Community health centerMobile testing unitCommunity centersSchools | Questionnaire/survey (in-person)Questionnaire/survey (online or by mail)Covid-19 Testing | 03/18/2021 | Phase 1 - First vaccine delivered and available to certain populations (between 12/14/20 and 04/19/21 |
| 91 | New York | Testing | African AmericanHispanic/Latin XLow income populations | -- | -- | Other community (non-clinical) settings study field site near NYU | Questionnaire/survey (in-person)Others- medical documentation of COVID tests provided by the participantOther qualitative interview | 06/30/2022 | Post Phase 4 - Vaccine available to all (after 05/23/22) |
| 92 | West Virginia | Testing | Rural Populations | Adults with substance useLower income populationsLow income housingTesting DesertsPeople Experiencing Homelessness | semi structured interview summariesCross-sectional cohort | Zoom videoconferencingSchools | InterviewCOVID-19 testing (choose this response if your project is administering COVID 19 testing) | 04/11/2022 | Phase 4 - First booster available to all (between 11/22/21 and 05/23/22) |
| 93 | Oregon | Testing | Adults with substance use | People Experiencing HomelessnessPeople living with HIV/AIDS (PLWHA) | Cross-sectional cohort | Community centers | Questionnaire/survey/(in-person)COVID-19 testing (choose this response if your project is administering COVID 19 testing) | 03/14/2022 | Phase 4 - First booster available to all (between 11/22/21 and 05/23/22) |
| 94 | Maryland | Testing | Hispanic/LatinXImmigrant | -- | Prospective observational cohort study | HomeOther community (non-clinical) settings - health fairs, churches, etc | Questionnaire/survey (in-person)Questionnaire/survey (online or by mail)Qualitative Focus Group | 08/08/2022 | Post Phase 4 - Vaccine available to all (after 05/23/22) |
| S11 | Missouri | Testing | African AmericanLow income populations | Low income housingPeople Experiencing Homelessness | Prospective observational cohort studyRandomized controlled trial | Home (e.g. mailed and email surveys, in-home interviews) | Questionnaire/survey (online or by mail) | 07/05/2022 | Post Phase 4 - Vaccine available to all (after 05/23/22) |
| S13 | Oregon | Testing | Hispanic / Latin XImmigrants | Rural populationFarm Workers | Prospective observational cohort studyRandomized controlled trial | Home (e.g. mailed and email surveys, in-home interviews)SchoolsCommunity centersMobile testing unitCommunity health center | Questionnaire/survey (in-person)Questionnaire/survey (online or by mail)COVID-19 testing (choose this response if your project is administering COVID 19 testing) | 09/15/2021 | Phase 2 - First vaccine available to all (between 04/20/21 and 09/25/21) |
| S14 | Arizona | Testing | Hispanic/LatinXAmerican Indians | ImmigrantsOlder AdultsRural PopulationsFarm WorkersCoal MinersLow income populationsTesting Deserts | Prospective observational cohort study | Home (e.g. mailed and email surveys, in-home interviews)Community centersOther community (non-clinical) settings-Parks, Health Fairs, Mexican Consulate, Door to door Outpatient clinic | Questionnaire/survey (in-person)Questionnaire/survey (online or by mail)COVID-19 testing (choose this response if your project is administering COVID 19 testing)Other biological data (e.g. blood sample, nasal swab) | 01/20/2022 | Phase 4 - First booster available to all (between 11/22/21 and 05/23/22) |
| S16 | Arkansas | Testing | Hispanic/LatinX | -- | Cross sectional cohort | Mobile testing unitCommunity centersOther community setting (community events-cultural events, holiday celebrations etc.) | Online questionnaire/surveyCovid-19 testing (choose thie response if your project is administering COVID 19 testing) | 03/18/2022 | Phase 4 - First booster available to all (between 11/22/21 and 05/23/22) |
| S17 | Maryland | Testing | Adults with substance use | Low income populations | Cross-sectional cohort | Mobile testing unit | Questionnaire/survey (in-person)COVID-19 testing (choose this response if your project is administering COVID 19 testing) | 02/22/2022 | Phase 4 - First booster available to all (between 11/22/21 and 05/23/22) |
| S21 | New York | Testing | African AmericanHispanic/LatinXAmerican IndiansAsianHawaiian / Pacific IslandersAlaskan NativeOlder AdultsLow income populationsLow income housingTesting Deserts | ImmigrantsLGBTQ/Sexual and Gender Minority (SGM)Pregnant WomenAdults with substance usePeople living with HIV/AIDS (PLWHA) | Cluster randomized trial | Home (e.g. mailed and email surveys, in-home interviews) | Questionnaire/survey (in-person)Questionnaire/survey (online or by mail)COVID-19 testing (choose this response if your project is administering COVID 19 testing) | 06/24/2022 | Post Phase 4 - Vaccine available to all (after 05/23/22) |
| S3 | Maine | Testing | Immigrant Low income populationPeople experiencing homelessnessother - uninsured | -- | Cross-sectional cohort Propsective observational cohort | Mobile testing unitCommunity health centerHome (e.g. mailed and email survyes, in-home interviews)Group homes (e.g. halfway house, nursing homes)Other-Emergency shelters, Housing First apartment buildings | Questionnaire/survey (in-person) Covid-19 testing (choose thie response if your project is administering COVID 19 testing) Questionnaire/survey (online) other | 01/14/2022 | Phase 4 - First booster available to all (between 11/22/21 and 05/23/22) |
| S4 | Georgia | Testing | African AmericanHispanic/Latin XRural populationsFederally Qualified Health Centers (FQHCs)Other-Had to have a diabetes diagnosis | -- | Other - quasi-experimental design using pre-post data collection | Home (e.g. mailed and email surveys, in-home interviews) | Questionnaire/survey (in-person)Questionnaire/survey (online or by mail) | 12/12/2022 | Post Phase 4 - Vaccine available to all (after 05/23/22) |
| S7 | Illinois | Testing | African AmericanFederally Qualified Health Centers (FQHCs) | African AmericanHispanic / Latin XImmigrants | Cross-sectional | Schools Community centersOther communnite (non-clinical) settingsOutpatient clinicHospitalCommunity health center | Questionnaire/survey (in-person)Questionnaire/survey(online or by mail)COVID-19 testing (choose this response if your project is administering COVID 19 testing)Other biological data (e.g. blood sample, nasal swab) | 02/17/2022 | Phase 4 - First booster available to all (between 11/22/21 and 05/23/22) |
